# Tea Plant Information Archive: a comprehensive genomics and bioinformatics platform for tea plant

**DOI:** 10.1111/pbi.13111

**Published:** 2019-04-11

**Authors:** En‐Hua Xia, Fang‐Dong Li, Wei Tong, Peng‐Hui Li, Qiong Wu, Hui‐Juan Zhao, Ruo‐Heng Ge, Ruo‐Pei Li, Ye‐Yun Li, Zheng‐Zhu Zhang, Chao‐Ling Wei, Xiao‐Chun Wan

**Affiliations:** ^1^ State Key Laboratory of Tea Plant Biology and Utilization Anhui Agricultural University Hefei 230036 China

**Keywords:** tea plant, knowledge database, comparative genomics, bioinformatics platform, evolution biology

## Abstract

Tea is the world's widely consumed nonalcohol beverage with essential economic and health benefits. Confronted with the increasing large‐scale omics‐data set particularly the genome sequence released in tea plant, the construction of a comprehensive knowledgebase is urgently needed to facilitate the utilization of these data sets towards molecular breeding. We hereby present the first integrative and specially designed web‐accessible database, Tea Plant Information Archive (TPIA; http://tpia.teaplant.org). The current release of TPIA employs the comprehensively annotated tea plant genome as framework and incorporates with abundant well‐organized transcriptomes, gene expressions (across species, tissues and stresses), orthologs and characteristic metabolites determining tea quality. It also hosts massive transcription factors, polymorphic simple sequence repeats, single nucleotide polymorphisms, correlations, manually curated functional genes and globally collected germplasm information. A variety of versatile analytic tools (e.g. JBrowse, blast, enrichment analysis, etc.) are established helping users to perform further comparative, evolutionary and functional analysis. We show a case application of TPIA that provides novel and interesting insights into the phytochemical content variation of section *Thea* of genus *Camellia* under a well‐resolved phylogenetic framework. The constructed knowledgebase of tea plant will serve as a central gateway for global tea community to better understand the tea plant biology that largely benefits the whole tea industry.

## Introduction

Tea is among the world's three most popular nonalcoholic beverages with important economic, health and cultural values (Xia *et al*., 2017). It is produced from the leaves of tea plant—a globally planted evergreen crop that belongs to the genus *Camellia* of family Theaceae (Mondal *et al*., 2004). Tea comprises plentiful characteristic metabolites such as tea polyphenol, caffeine, amino acids (mainly theanine), vitamins and minerals that are beneficial to human health (Higdon and Frei, 2003; da Silva, 2013; Yang and Landau, 2000). Currently, tea plant has been introduced to more than 50 countries around the world for large‐scale commercial cultivation. Over three billion people drink tea in more than 160 countries. According to the statistics from the Food and Agricultural Organization of the United Nations (FAO; www.fao.org/faostat/), the globe planting area of tea plant has exceeded 4.1 million hectares, and more than 5.95 million metric tons of tea worldwide are annually produced.

The development of crop genomics has played an important role in the effective use of modern molecular biology towards crop genetic improvement (Morrell *et al*., 2012). Particularly in the last decade, the sequencing and/or resequencing of several important crops such as rice (Huang *et al*., 2010), wheat (Cavanagh *et al*., 2013), corn (Tian *et al*., 2011), soybean (Li *et al*., 2014), cotton (Zhang *et al*., 2015b) and vegetables (Liu *et al*., 2014; Qi *et al*., 2013) has essentially promoted the large‐scale cloning and identification of abundant genes associated with important agronomic traits. This greatly accelerated the generation of new varieties with increased yields and quality. However, tea is the oldest and the world's most popular nonalcohol beverage with significant economic and medicinal importance; the lacking of reference genome basically hampers the urgent needs to utilize this precious genetic resource towards modern molecular breeding. To address this, we spend more than 10 years to generate a high‐quality reference genome of tea plant using two most advanced sequencing technologies, including PacBio long‐read and Illumina paired‐end sequencing (Wei *et al*., 2018). Using the genomic, phylogenetic, transcriptomic and phytochemical approaches, we identified and functionally validated a key gene involving in the biosynthesis of theanine, an important characteristic compound associated with tea quality, and meanwhile showed strong evidences that the unique properties associated with tea cultivation and selection are the products of a series of dynamic genome evolutionary events. This broadens our understanding of the genetic basis of biosynthesis of the major characteristic secondary metabolites of tea plant that determines the tea quality and thus would promote the germplasm utilization for breeding new improved tea varieties (Wei *et al*., 2018).

Facing with the huge biological data concomitantly generated during the genome sequencing and widely published by other tea researchers, how to effectively integrate and share them with the tea community to speed up the resolving of major biological questions associated with tea industry is currently the main problem. We thus here built the first dynamic, interactive and scalable tea plant genome database (TPIA), which contains the complete cultivated tea plant genome (cultivar shuchazao) and widely collected transcriptomes, metabolomes and representative germplasm resources worldwide. A large number of versatile analytic tools (e.g. blast, correlation analysis and expression differential analysis) are launched to establish their internal relationships. These data resources will freely serve the entire tea research community and will be of significance for the future genetic engineering, functional genomics and population genetic studies in tea plant.

## Data content

### Genomics data

#### Whole‐genome sequence data

The current release of tea plant genome (*Camellia sinensis* var. *sinensis* cv. shuchazao) is 3.14 Gb (2.89 Gb without N) that consists of 14 051 scaffolds and 94 321 contigs (Wei *et al*., 2018) (Table 1). The contig and scaffold N50 is 67.07 kb and 1.39 Mb, respectively. Average GC content is 37.84%. The maximum length of contig and scaffold is 538.75 kb and 7.31 Mb, respectively.

**Table 1:** Data resources in TPIA database (as of 1 January 2019)

Data type	Entries	Data type	Entries
**Genome**		**Transcriptome**	
Assembly	1	Assembly of tea plant	60
Gene models	33 932	PacBio SMRT transcriptome	1
Blast to CSA (Yunkang 10)	32 708	Expression studies (developmental stages)	8
Blast to *A. thaliana* (version 10)	32 122	Expression studies (under biotic and abiotic stresses)	5
Annotated to NR database	33 380	Assemblies from close relatives	23
Annotated to PFAM database	27 262	Polymorphic EST‐SSR	1663
Annotated to GO database	15 138	SNP genetic variations	190 363
Annotated to KEGG pathway	27 267	**Metabolite**	
Annotated to KOG groups	19 230	Catechins	26
Annotated to COG groups	13 757	PAs	25
Annotated to InterPro database	27 262	Theanine	26
Transposable elements (Gb)	1.86	Caffeine	25
Transcription factors	2486	**Germplasm resource**	
Simple sequence repeat (SSR)	59 765	Wild	37
OrthMCL gene family	15 224	Breeding varieties	352
Organelle genome/insertions	19/269	Other lines	731
Experimentally validated genes	107	Total	1120
Genomic synteny	151	**Small RNAs**	
**DNA methylation**		CircRNA	1942
Leaves	2	microRNA	767

#### Gene models

The current tea plant genome assembly hosts a total of 33 932 high‐confidence genes (Wei *et al*., 2018) (Table 1). They are functionally annotated using different databases, including NR database (33 380; 98.37%), COG database (13 757; 40.54%), KOG database (19 230; 56.67%), PFAM database (27 262; 80.34%), GO database (15 138; 44.61%), KEGG database (27 267; 80.36%), InterPro database (27 262; 80.34%), TAIR database (32 122; 94.67%) and the best hits in *C. sinensis* var. *assamica* cv. Yunkang 10 gene sets (32 708; 96.39%) (Table 1).

#### *Bacterial artificial chromosome* (*BAC*) *end sequence*

A total of 20 complete BAC clones and 738 BAC end sequences (BESs) of tea plant, ranging from 105 to 917 bp, were collected from Tai *et al*., 2017; (Tai *et al*., 2017). The average BAC insert length is around 113 kb. The total length of the 738 BESs was 501 459 bp with an average read length of 679.48 bp after trimming the vector sequence.

#### Transposable elements

The combined *de novo* and homology‐based methods were used to detect transposable elements (TEs) in the tea plant genome (Wei *et al*., 2018). This identified a total amount of 1.86 Gb TE sequences that covers 64% of the nongapped tea plant genome assembly (Table 1). The long terminal repeat (LTR) elements were the dominating TE type, accounting for 90.8% of all TEs and 58.6% of the total assembly. Of the LTR retrotransposons, the *Gypsy*‐ and *Copia*‐type are the two chief ones, covering ~45.85% and ~8.24% of the genome assembly, respectively.

#### Transcription factors and simple sequence repeats

A total of 2486 (7.32% of all the protein‐coding genes) transcription factor (TF) genes were identified and classified into 67 families using the iTAK package (Zheng *et al*., 2016) (Table 1). The simple sequence repeats (SSRs) were identified using the pipeline developed in Wei *et al*., 2018; (Wei *et al*., 2018) (Table 1). This resulted in a total of 59 765 SSRs in the genome assembly.

#### Orthologous/paralogs gene families

The OrthoMCL pipeline (Li *et al*., 2003) (version 1.4) that integrated BLAST alignment (1e‐5) and Markov cluster algorithm (MCL; *I* = 1.5) was used to identify orthologous and paralogous gene families between tea plant and other 11 representative plant genomes, including grape (Jaillon *et al*., 2007), poplar (Tuskan *et al*., 2006), coffee (Denoeud *et al*., 2014), cocoa (Argout *et al*., 2011), African oil palm (Singh *et al*., 2013), peach (Verde *et al*., 2013), Medicago (Young *et al*., 2011), kiwifruit (Huang *et al*., 2013), *Amborella* (Albert *et al*., 2013), *Arabidopsis* (Initiative, 2000) and Assam tea (Xia *et al*., 2017). In total, 27 610 genes in 15 224 gene families were identified (Table 1).

#### Genome Synteny

We identified a total of 151 genome syntenic blocks between/within Assam tea and Chinese tea using MCScanX based on their gene annotation results. It should be pointed out that the total number of identified blocks is underestimated. The current release of tea plant genomes are still in draft, and consist of several short sequences that affect the precise detection of the genome collinearity.

#### Organelle genomes and DNA insertions

The organelle genomes from tea plant and close relatives were collected from NCBI database. The current collection contains 19 chloroplast (cp) genomes with detail gene annotations and related reference information (Table 1). The insertions (>1 kb) of organelle genome to nuclear genome were identified using BLAST with a threshold E‐value <1e^−5^ and identity >0.9 (Altschul *et al*., 1997). This harvests an average of 269 chloroplast insertions distributed in 141 scaffolds of tea plant genome assembly.

#### Function experimentally validated genes

A total of 107 experimentally validated genes of tea plant were collected from published literatures that cover major research hotspots of tea plant in the last decades (Table 1). We manually classified them into four functional categories, including secondary metabolism, signalling, abiotic and/or biotic stress, and development. The locations of these genes in the tea plant genome assembly were determined using BLAST and further visualized using JBrowser (Skinner *et al*., 2009). The basic information for these genes, including study background, authors, abstract, representative figures, cloned protein and gene sequences, and functional category, was collected and automatically linked to the NCBI database and published journals.

### Transcriptomic data

#### Transcriptomes of tea plant from different tissues

A total of 94.11 Gb RNA sequencing (RNA‐seq) data were acquired from eight representative tissues of tea plant, including apical buds (AB), young leaves (YL), mature leaves (ML), old leaves (OL), immature stems (ST), flowers (FL), young fruits (FR) and tender roots (RT) (Wei *et al*., 2018) (Table 1). Expression profiles of all the 33 932 tea plant protein‐coding genes were calculated by mapping the sequenced RNA‐seq data to the genome assembly and evaluated using transcripts per million (TPM). Additionally, we generated a total of 361 947 reads from four libraries (0~1 Kb, 1~2 Kb, 2~3 Kb and 3~6 Kb) using PacBio SMRT sequencing platform (Xu *et al*., 2017). This generated 80 217 transcripts with an average length of 1781 bp (Table 1).

#### Transcriptomes of tea plant under various biotic and abiotic stresses

Transcriptomes of tea plant under diverse biotic and abiotic stresses were collected from publicly available database. (i) Cold tolerance: approximately 57.35 million (5.16 Gb) RNA‐Seq reads were collected from leaves of tea plant at three stages of cold acclimation (CA) process, including nonacclimated (CK), fully acclimated (CA1) and de‐acclimated (CA3) (Wang *et al*., 2013). To further depict the landscape of cold tolerance in tea plant, we also generated a total of 161 Gb RNA‐seq data from five stages of tea plant during cold acclimation, including nonacclimated at 25~20 °C (CK), fully acclimated at 10 °C for 6 h (CA1‐6 h) and 10~4 °C for 7 days (CA1‐7d), cold response at 4~0 °C for 7 days (CA2‐7d) and recovering under 25~20 °C for 7 days (DA‐7d). (ii) Drought tolerance: a total of 108.82 million RNA‐seq reads were collected from young leaves of tea plant subjected to continuous drought stress, including four stages: 25% polyethylene glycol (PEG) treatment for 0, 24, 48 and 72 h (Zhang *et al*., 2017). The total length of the sequencing data is 5.5 Gb. (iii) Salinity stress: a total of 149.48 million RNA‐seq reads (7.55 Gb) were collected from leaves of tea plant under salt stress (Zhang *et al*., 2017). The 200 mm NaCl was used to simulate salt‐stress conditions for tea plant with 0, 24, 48 and 72 h. (iv) methyl jasmonate (MeJA) response: RNA‐seq data from leaves of tea plant in response to MeJA were collected from Shi *et al*. (2015). In total, more than 27 million 100‐bp paired‐end reads were produced from four samples treated using MeJA for 0, 12, 24 and 48 h (Table 1). All data sets were preprocessed to remove adapter, low‐quality base and potential contaminations. The remaining high‐quality reads were then aligned to tea plant genome assembly to calculate the expression levels with further differential expression test. Transcripts per million (TPM) were used to evaluate expression level.

#### Transcriptomes collected from diverse tea plant close relatives

Beside the transcriptomes of tea plant collected from different developmental stages or under different stresses or response to different hormones, we also literately gathered a total of nine transcriptomes from its close relatives, including *C. azalea* (Fan *et al*., 2015), *C. japonica* (Li *et al*., 2016), *C. meiocarpa* (Feng *et al*., 2017), *C. nitidissima* (Zhou *et al*., 2017), *C. oleifera* (Xia *et al*., 2014), *C. reticulate* (Yao *et al*., 2016), *C. sasanqua* (Huang *et al*., 2017), *C. chekiangoleosa* (Wang *et al*., 2014) and *C. taliensis* (Zhang *et al*., 2015a). The detail information of each transcriptome that comprises published journal, authors, study background, main conclusion, data accession numbers and investigated tissues was collected. The trinity software (Grabherr *et al*., 2011) was used to assemble the sequencing data into transcripts. To further expand the gene pool of tea plant, we also additionally sequenced and assembled the leaf transcriptomes of 13 *Camellia* species that nearly covers all species from Section *Thea* of genus *Camellia*. They are *C. jingyunshanica*,* C. makuanica*,* C. atrothea*,* C. pubescens*,* C. tachangensis*,* C. parvisepala*,* C. kwangsiensis*,* C. angustifolia*,* C. ptilophylla*,* C. leptophylla*,* C. gymnogyna*,* C. crassicolumna* and *C. tetracocca*. The transcriptomes publicly collected from tea plant and its close relatives offer an essential genetic resource for the future tea plant genetic improvement and comparative genomics studies.

#### *Polymorphic EST‐SSRs* (*PolySSR*)

The CandiSSR pipeline (Xia *et al*., 2016) was used to identify PloySSRs between tea plant and other 19 representative *Camellia* species. In total, 1663 polymorphic EST‐SSRs were identified. To assist the marker development, three primer pairs for each identified PolySSR (totally 4989) were further designed. To the best of our knowledge, although there are much more SSRs previously reported in the genus *Camellia*, relatively fewer polymorphic loci have been identified (Ma *et al*., 2010; Tong *et al*., 2013). Thus, the PolySSRs reported here will be of particularly valuable for the germplasm characterization and population genetic studies of tea plant in the future.

#### Homologs of Camellia transcript in the tea plant genome

The homologs of each *Camellia* transcript in the tea plant genome were determined by aligning them against the tea plant genome assembly using BLAST (Altschul *et al*., 1997). Only the best hit was retained.

### Metabolites data

#### Catechins

The content of catechins in eight representative tissues of tea plant, including AB, YL, ML, OL, ST, FL, FR and RT, was determined using high‐performance liquid chromatography (HPLC) system (Wei *et al*., 2018). In total, six types of catechins were detected. They are (+)‐catechin (C), (−)‐epicatechin (EC), (+)‐gallocatechin (GC), (−)‐epigallocatechin (EGC), (−)‐epicatechin‐3‐gallate (ECG) and (−)‐epigallocatechin‐3‐gallate (EGCG). The total contents of catechins in eight tissues varied, ranging from tender roots (0.68 mg/g dry weight) to apical buds (212.32 mg/g dry weight), with an average of 91.46 mg/g dry weight in each tissue (Table 1). The catechin contents in leaves of 25 close relatives of tea plant were also collected (Xia *et al*., 2017).

#### Polymer proanthocyanidins

The polymer proanthocyanidins (PAs) in eight tissues of tea plant were detected using the method described previously (Pang *et al*., 2008). In total, an average of 96.38 mg/g dry weight PAs for each tissues were determined. The PAs were further classified into soluble and nonsoluble PAs using DMACA‐staining methods. The average content of soluble PAs in each tea plant tissue was 77.16 mg/g dry weight, much higher than the content of nonsoluble PAs (19.22 mg/g dry weight) (Table 1).

#### Theanine

The contents of theanine in eight tissues of tea plant were determined using HPLC method (Wei *et al*., 2018). This detected an average of 14.89 mg/g dry weight theanine in tea plant tissues. Root harboured the highest content of theanine. No obviously theanine was detected in mature and old leaves. The theanine contents were also collected from leaves of 25 close relatives of tea plant (Xia *et al*., 2017).

#### Caffeine

The caffeine content in leaves of tea plant and 25 close relatives was collected (Xia *et al*., 2017). All collected data were deposited into TPIA.

### Correlation data

#### Gene co‐expression data

The co‐expression network of 33 932 tea plant genes among eight different tissues and under various biotic or abiotic stresses (cold, drought, salt and MeJA) was constructed using three methods (Pearson, Spearman and Kendall) implemented in R platform. Only the connection between genes with PCC value cut‐off of ≥|0.75| and a *P*‐value ≤0.05 was retained and regarded as expression correlated.

#### Correlation between gene expression and metabolite accumulation

The correlation between gene expression and metabolite accumulations was built based on the metabolite data and gene expression data using Pearson, Spearman and Kendall methods. Similarly, the correlations with PCC ≤ |0.75| and *P*‐value ≥0.05 were filtered.

### Genetic variation data

A total of 190 363 high‐quality single nucleotide polymorphisms (SNPs) were identified from 16 close *Camellia* species that diverged from their common ancestor ~4 million years ago (MYA) (Table 1). These species covered nearly all tea plant species from Section *Thea*, an important *Camellia* group particularly helpful for tea plant genetic improvement. Leaf RNA‐seq data were first aligned to tea plant genome assembly using BWA package (Li and Durbin, 2009), and the alignments were then fed to GATK pipeline to discover SNPs (McKenna *et al*., 2010). The SNPs with missing data or minor allele frequency (MAF) <0.05 were removed. JBrowse (Skinner *et al*., 2009) was used to visualize the detected SNP variants.

### DNA methylation and noncoding RNA data

The DNA methylation data were collected from young leaves of tea plant (Wang *et al*., 2018). In total, approximate 205.5 Gb raw sequencing reads were downloaded (SRR7832302 and SRR7832303) and preprocessed to mapping against the tea plant genome using BSMAP (Xi and Li, 2009). The ratio and types of methyl‐cytosines were identified by ‘*methratio.py*’ script implemented in BSMAP. The circRNA data were obtained from leaf of tea plant (Tong *et al*., 2018). The miRNA data were collected from different cultivars and experiments. In total, 1.2 Gb and 289.4 Mb data were separately gathered from young leaves of cultivar 1005 and shuchazao, while 452.3, 551.3, 372.2, 372.4, 452.3 Mb sequencing data were harvested from tea plant during cold acclimation (control and treatment), cold stress (4 °C) for 4 and 8 h, and 25 °C for 4 h, respectively. The clean sequencing data were applied to discover known and novel miRNAs using miRDeep2 (Friedlnder *et al*., 2011), generating a total of 767 microRNAs with an average of 110 for each sample.

### Germplasm data

The information of >1100 tea plant germplasm was collected from 17 provinces of China and 13 regions of 10 other tea producing countries. This includes 37 (3%) of wild resources (*C. taliensis*), 352 (31%, including 268 Chinese elite lines) of breeding varieties and 731 collected germplasm (Assam type and China type).

### Downloads

Genome assembly (FASTA), gene prediction (GFF3), gene functional annotations (TSV), transposable element (GFF3), organelle genome data (FASTA), experimentally validated gene data (FASTA), gene family data (TXT), transcription factor data (XLS), transcriptomic data (FASTA), polymorphic EST‐SSR data (XLS), metabolite data (XLS), co‐expression data (XLS), orthologous groups data (TXT), gene expression data (XLS), SNP data (VCF), germplasm resource data (XLS), DNA methylation data (BED), circRNA and microRNA data (BED) and other related data can be downloaded at http://tpia.teaplant.org/download.html.

## Facilities and tools

### Implementation

**Figure 1:**
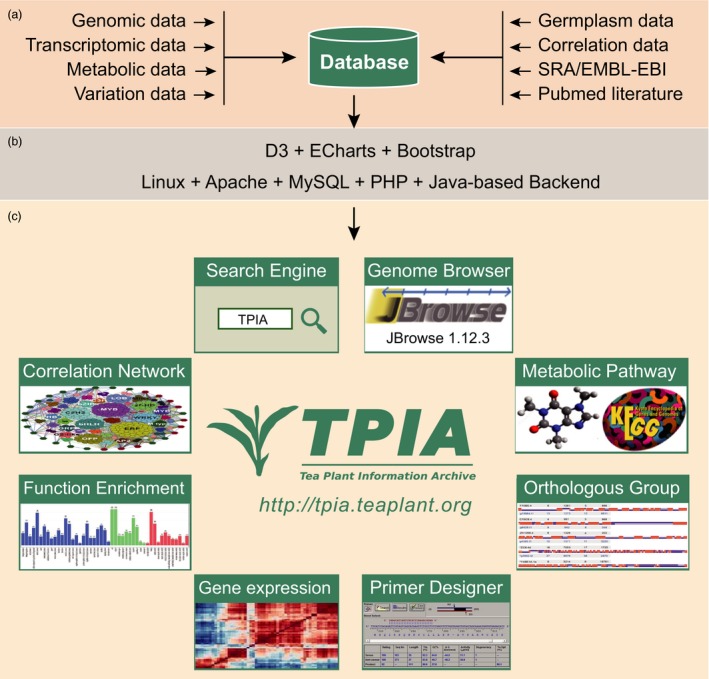
Architecture of TPIA database. (a) Data source layer; (b) middleware layer; (c) application layer.

We implemented TPIA using three major software that include MySQL database, Apache Tomcat web server and Java‐based computational toolkits (Figure 1). The omics‐data sets and their relevant resources are restored in Linux platform with MySQL database. The data sets include genomic data, transcriptomic data, metabolic data, variation data, germplasm data, correlation data and related source data (e.g. NCBI SRA data and PubMed literatures). The web services are established using Apache web server, a popular and widely used application supporting multiple plug‐ins that benefit the server enhancement. Data manipulation and visualization are mainly implemented using JavaScript library Data‐Driven Documents (D3), Bootstrap, Perl, R scripts and Echart, which is a web‐based and cross‐platform framework for rapid data visualization. Google Chrome and IE 9.0+ are preferred to achieve the best display effect. TPIA is available at http://tpia.teaplant.org.

### Genomic views using JBrowse

One key mission of TPIA is to integrate and annotate large‐scale omics‐data sets from published experiments on tea plant and share them with the global tea researchers to facilitate the development of tea industry. To achieve this goal, we set up JBrowse, a fast and interactive genome browser widely used for navigating large‐scale high‐throughput sequencing data under a genomic framework (Skinner *et al*., 2009). JBrowse is highly flexible and customizable. It also allows users to load their own sequence data sets for visualization and comparisons with data sets in TPIA.

**Figure 2:**
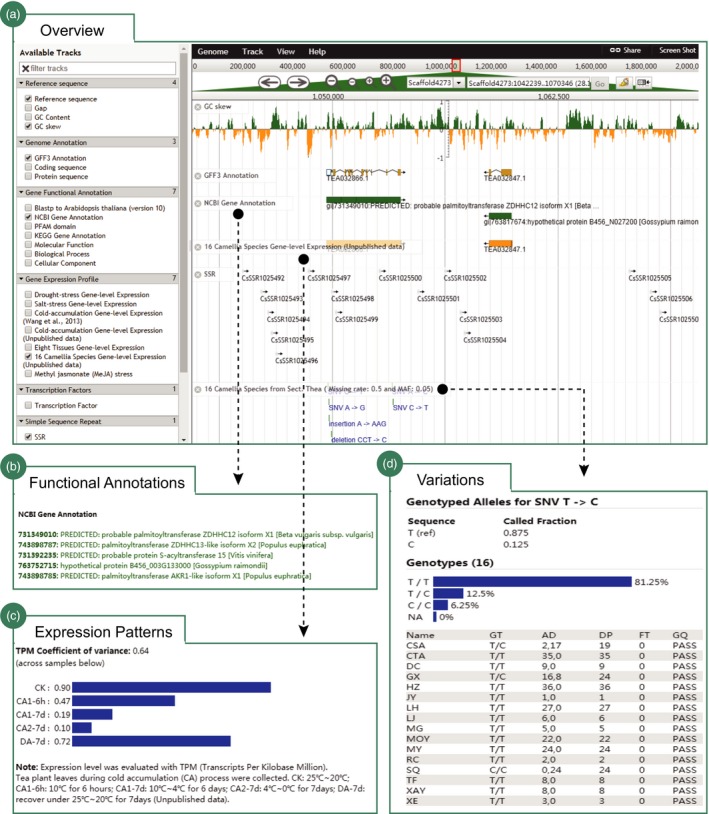
Visualization of the major genomic features of tea plant using JBrowse. (a) Overview of the JBrowse that totally includes 27 tracks; (b) functional annotations of tea plant genes using NCBI NR database (totally seven annotations); (c) expression patterns of tea plant genes (e.g. cold acclimations) at different tissues or under stress or in leaves of other *Camellia* species; and (d) variations of tea plant genes among 16 representative *Camellia* species.

The current TPIA offers the latest assembly of tea plant genome for viewing. It includes 27 genomic features (Figure 2a). Users can select specific tracks to view them, including base‐level of reference sequence, gap sequence, GC content, transposable elements, transcription factors, simple sequence repeats, gene models, functions assigned using PFAM domain, KEGG pathway, GO category and blast matches of putative orthologous genes from NCBI NR database and *A. thaliana* (version 10). The majority of the presented features were clickable and will be linked to a window that shows the detail information about the selected features (Figure 2b). Cross‐linking to public databases for some features was available. We provide abundant high‐throughput RNA sequencing (RNA‐Seq) data sets from plentiful expression experiments on tea plant (Figure 2c). The principal tracks highlighted the gene expression profiles of tea plant genes under various biotic and abiotic stresses, including cold acclimation, drought tolerance, salinity stress and methyl jasmonate (MeJA) response. We also offered available RNA‐seq data sets and expression data sets generated from a total of eight tissues that cover nearly all developmental stages of tea plant, including AB, YL, ML, OL, ST, FL, FR and RT. In addition, we incorporate RNA‐seq data sets from 16 close tea plant relatives: gene expression in leaves, and genetic variation data (SNP) between tea plant and these 16 close relatives (Figure 2d). The tracks allow users to intensely visualize and explore SNP variations and their effects on tea plant genes. We will add more novel and publicly available data types and analysis tracks to JBrowse as they were available.

### Search engine

**Figure 3:**
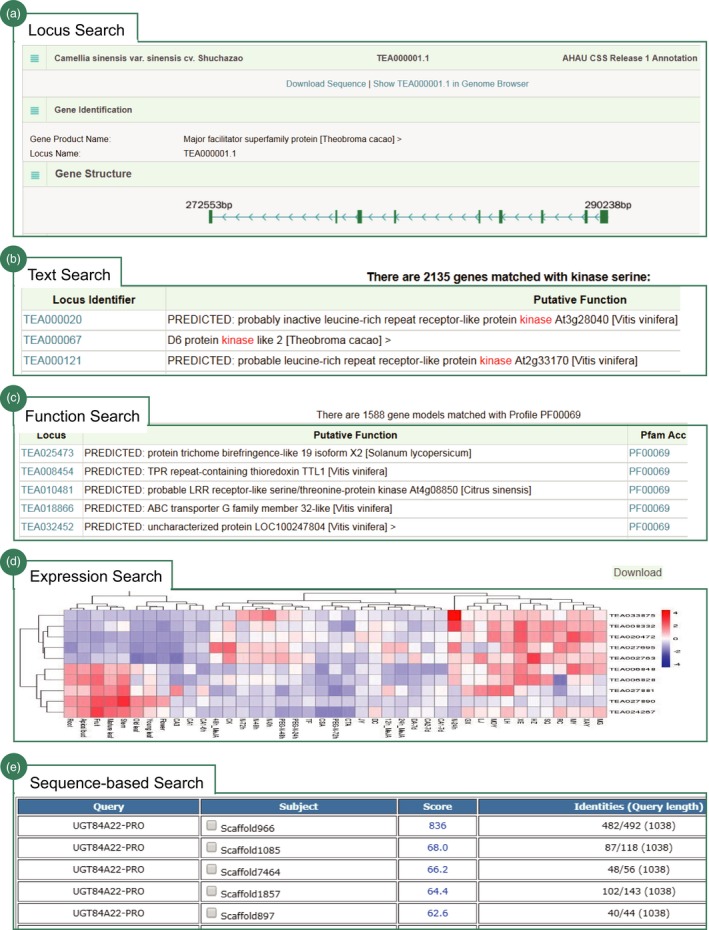
Search engine of TPIA database. Screenshot of the results from (a) locus search (e.g. TEA000001.1); (b) text search (e.g. kinase); (c) function‐based search (e.g. PF00069, protein kinase domain); (d) search expression patterns for 10 genes; and (e) sequence‐based search (e.g. UGT84A22).

We designed a powerful search engine helping users to deeply retrieve and graphically visualize the data in TPIA. It mainly includes five flexible search options. Users can use the gene identifier, keyword, function property, expression pattern and sequence to search TPIA. (i) Gene identifier search: users can use a gene locus identifier to quickly search TPIA. The response is a dynamic table that summarizes the details of searched gene, including genomic location, gene structure, functional category (GO, KEGG, PFAM, Interpro), homologs in other plant species, nucleotide and amino acid sequences, and expression pattern at different developmental stages or under diverse biotic and abiotic stresses or in leaves of different close tea plant species (Figure 3a). The majority of these results are clickable and can be graphically illustrated on the genome browser implemented using JBrowser (Skinner *et al*., 2009) or cross‐linked to specific external databases. The output can be also downloaded as FASTA file for further analysis using Galaxy. (ii) Text search: users can use keywords to massively search information in TPIA. The response returns all genes associated with the search items that include gene identifiers and putative functions (Figure 3b). Similarly, the gene identifiers are clickable and can be linked to JBrowser for visualization. Results can be also batch downloaded as TXT file for publication or further analysis. (iii) Function‐based search: users can use four functional categories (GO, KEGG, PFAM and Interpro) to search genes in TPIA. The responses are a set of genes annotated with the examined functions (Figure 3c). Details of the genes include clickable gene identifiers, putative functions, cross‐linked accession number and description in other external databases. (iv) Expression search: users can input a list of gene identifiers of interest to search their expression patterns under stress or at different tissues of tea plant or in leaves of close relatives. The output is a heatmap that graphically shows the expression level and can be downloaded locally for further analysis (Figure 3d). (v) Sequence‐based search: largely different from the above search strategies that focus on searching TPIA using gene identifiers, keywords or gene properties, the sequence‐based search was a new method designed to find homologs in tea plant genome using diverse biological sequences. The output supports multiple formats (flat, xml, tabular, etc.) that can be bulk downloaded as FASTA or Excel files for advance analysis (Figure 3e). All the above search methods are finally integrated to build an advanced search tool with multiple filter options.

Overall, the search engine provides users an easy, versatile and web‐accessible tool to systemically retrieve the abundant of genetic data of tea plant in TPIA, which will be of great importance for future functional genomics and genetic improvement efforts in tea plants.

### Analysis of tea plant genes

**Figure 4:**
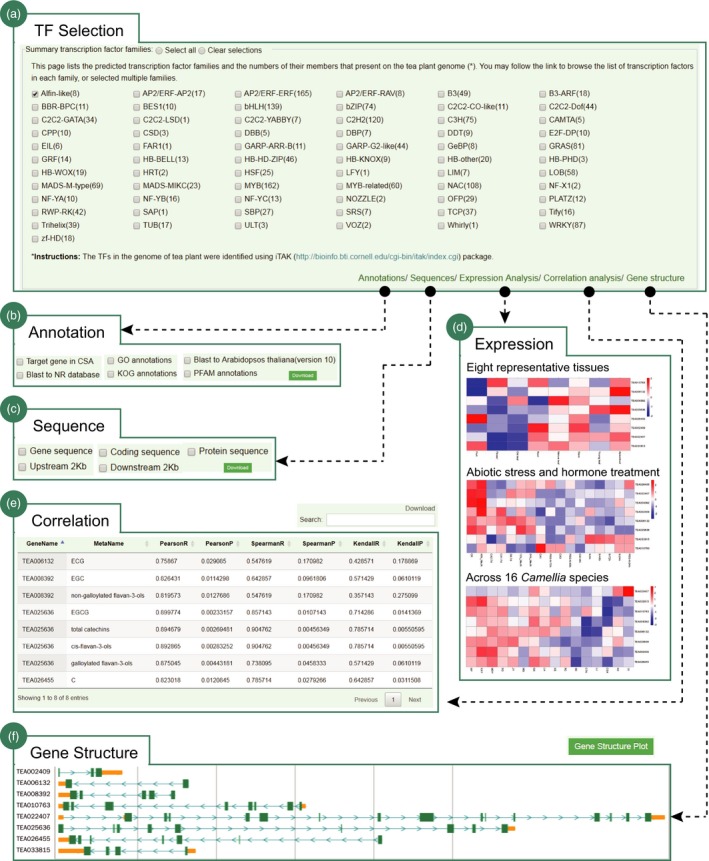
Genomewide analysis of tea plant transcription factors. (a) A total of 2486 TFs from 67 families are provided for analysis; a case study for Alfin‐like gene family, including (b) functional annotation, (c) multiple sequence download, (d) expression patterns at different tissues, or under diverse abiotic stresses (cold, drought, salt) and hormone treatment (MeJA), or across 16 *Camellia* species, (e) correlation with genes and metabolites and (f) gene structure visualization.

We have systematically annotated the tea plant genome and made them, particularly those essential genomic function elements, easily accessible for searching and analysing by researchers. For example, we establish an interactive interface to assist users to genomewidely investigate the tea plant transcription factor (TF), an important regulator in both plant development and stress response. In total, 2486 TFs from 67 families are supplied. The users can simultaneously select one or more TFs for analysis (Figure 4a). We provide five options for downstream analysis, including functional annotation, expression analysis, correlation analysis, sequence extraction and gene structure visualization (Figure 4b–f). The output can be downloaded for further function experimental validation and regulation mechanism investigation. We provide a web portal for users to search genomic SSRs of tea plant with an option to further exam their polymorphism among the 14 close relatives (Figure S1). The user can select a specific SSR type (di‐ to hexamer) or enter a definite SSR motif to search. The output is a table that shows the details of returned SSRs, including SSR identifier, genomic location, SSR type, SSR motif, SSR size and polymorphic status. The upstream and downstream 100‐bp sequence, and three primer pairs for each SSR were output and can be downloaded to perform further population genetic studies. Besides, we designed an interface helping users to retrieve the repeat elements of tea plant (Figure S2). It supports three searching options, including annotation methods, repeat types and genomic locations. The results are the details of searched repeats that can be downloaded for further functional and evolutionary analysis. In addition, we manually collected nearly all cloned genes from the published literatures and designed a flexible web portal to make them easily searchable for users (Figure S3). The users can search genes by species name or function category (e.g. metabolism, signalling, development, and biotic and abiotic stress). The output is a table which shows the details of the cloned genes, including gene symbol, species name, cultivar, NCBI accession number, gene length, functional description and related references. The representative figures showing the experimental validation of gene functions are also provided. Moreover, various types of organelle insertions can also be retrieved, visualized and downloaded for further functional and evolutionary analysis.

### Collection and utilization of tea plant transcriptomes

We have read the almost entire available published scientific literatures describing the transcriptome sequencing experiments on tea plant and close relatives (as of August 2018). In total, 60 transcriptomes from 26 tea plant cultivars and 21 close relatives were collected and integrated into TPIA (Figure S4A). To provide users a landscape of tea plant transcriptome, we selectively *de novo* assembled the data from two tea plants and seventeen representative close species into transcripts using Trinity. This generated a total of 2 283 723 transcripts with 114 186 for each species, representing the most comprehensive tea gene pool to date (Figure S4B). All the assembled transcript data sets are well organized and can be batch downloaded directly from TPIA for comparative studies. We also annotated the putative functions of the assembled transcripts by carefully aligning them against various well‐known public protein databases. Users can retrieve the function by species name. The responses are functional details of transcripts assigned by SWISS‐PROT, PFAM, GO, KEGG pathway, InterPros, KOG and COG, which can be downloaded as Excel files (Figure S4C). The homologous proteins for each transcript were clickable and can be cross‐linked to specific public database. Polymorphic EST‐SSR (PolySSR) is among the most important and broadly used molecular markers. It is derived from transcribed gene regions and thus particularly important for the future plant breeding. We provided a total of 1663 EST‐SSRs of tea plant in TPIA; they all exhibit polymorphism among 20 tea plant species/varieties (Figure S4D). To facilitate data extraction, we provided four options that include SSR type, missing rate, standard deviation and primer transferability. The results described the details of searched SSR, including SSR type, genomic location, polymorphism among 20 tea plant species, three candidate primer pairs and flanking 100 bp for each searched PolySSR. Besides, we made a total of 43 158 TFs from 20 tea plant species/varieties available in TPIA to accelerate the exploration of TFs in tea plant (Figure S4E). Users can search the specific TF of interest by species name and TF type. In response, the detail information for each searched TF, including transcript ID, TF type/family, and EST and protein sequence, was returned and can be downloaded locally for further analysis. Finally, we build the relationships between tea plant genome and the assembled *Camellia* transcripts using BLAST. A web form allows the users to input a specific gene locus identifier of interest to search TPIA (Figure S4F). The output is a dynamic table that list the homologous transcripts in other 20 close species with comprehensive annotations.

### Visualization of tea quality‐related metabolites and pathways

**Figure 5:**
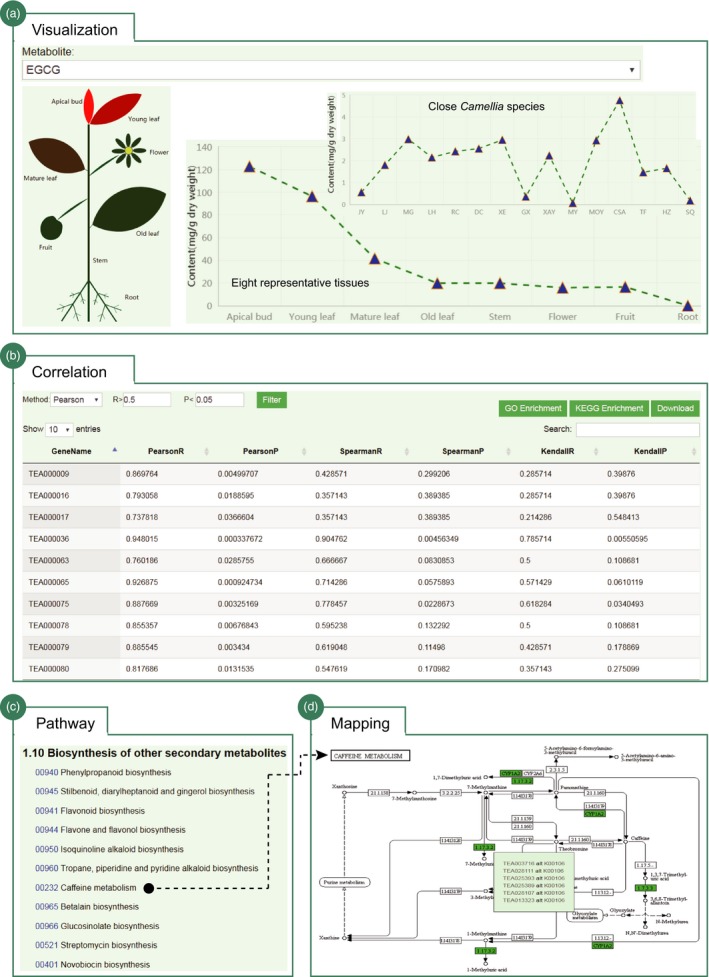
Visualization of tea quality‐related metabolites and pathways. (a) Visualization of the EGCG accumulations in eight representative tissues of tea plant and other 15 *Camellia* species; (b) the genes whose expression patterns are highly correlated with the accumulation patterns of selected metabolites (e.g. EGCG); further filtering options, analysis tools and download menus are provided; (c) biosynthesis pathways of secondary metabolites; and (d) pathway mapping for caffeine metabolism; the tea plant genes in the pathway are highlighted in green and cross‐linked to JBrowser and KEGG database (https://www.genome.jp/kegg/).

Tea plant is rich in secondary metabolites that determine the tea quality. We provided an interactive interface for users to retrieve, visualize and investigate the catechins and polymer proanthocyanidins (PAs) accumulations in eight representative tissues of tea plant (Figure 5a). In total, six major types of catechins that include C, EC, GC, EGC, ECG and EGCG and two types of PAs (soluble and nonsoluble PAs) are presented. Users can select specific metabolites from the pull‐down menu to view its accumulations in eight representative tissues of tea plant. The genes with expression patterns highly correlated with the accumulation pattern of specified metabolites are computationally predicted (Figure 5b). We provide a total of three correlation options helping users to perform further filtering. The results can be bulk downloaded or linked to analytic tools to perform functional enrichment analysis. Besides, we also collected the accumulation data of three major characteristic metabolites (catechins, theanine and caffeine) in leaves of tea plant and 15 close relatives (Figure 5a). Their accumulation patterns are illustrated as line plot. Similarly, the genes with expression patterns correlated with the accumulation pattern of selected metabolite are characterized and can be further filtered and downloaded to perform additional analysis (e.g. enrichment analysis and expression analysis). We also generated the metabolic pathways of tea plant genes using the annotated KEGG orthologs (Figure 5c). Users can click the specific metabolic pathways of interest (e.g. caffeine metabolism) to globally observe the gene distributions in tea plant genome. The enzymes/proteins that have KEGG orthologs in tea plant are demonstrated in green and cross‐linked to JBrowser and KEGG database (https://www.genome.jp/kegg/) (Figure 5d).

### Tea plant germplasm information system

Tea germplasm resources are valuable fundamental materials for tea plant breeding and biotechnology. Therefore, the construction of the tea plant germplasm information sharing platform is of great significance and can promote the integration, protection and utilization of germplasm resources that benefit the whole tea industry. In the current release of TPIA, we have collected and stored the information of >1100 tea plant germplasm from 17 provinces of China and 13 regions of 10 other countries (Table 1). This includes 37 (3%) of wild resources (*C. taliensis*), 352 (31%, including 268 Chinese elite lines) of breeding varieties and 731 collected germplasm (Assam type and China type). An interactive interface was designed for users to retrieve and visualize the location and related genetic data sets (Figure S5A). Users can search the germplasm of interest by species morphotype and/or country (region) location. The output is visualized using world map. Users can click the relevant parts of the world map to check details of searched germplasms, including accession number in TPIA, voucher number in KUN (http://kun.kingdonia.org/), species name, morphotype, locations (origin, latitude and longitude) and related reference (Figure S5B‐D). We also provided an option to allow users to further exam whether the marker sequences (e.g. SSR and cpDNA) available or not for the searched germplasm.

### Additional tools for customized analyses

**Figure 6:**
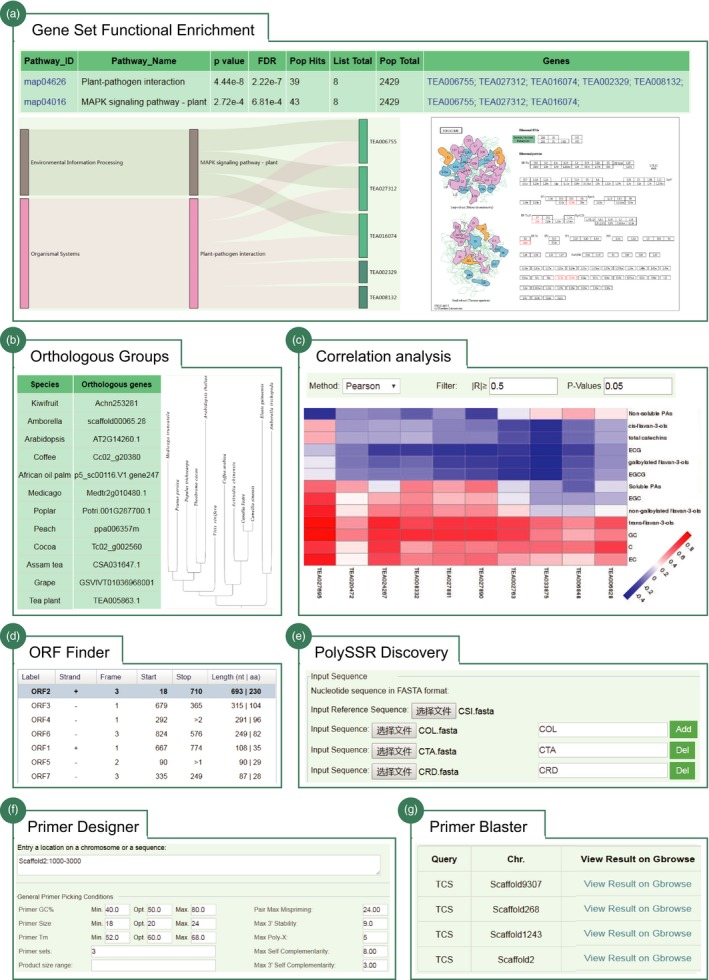
Flexible tools implemented in TPIA. (a) Gene set functional enrichment analysis using KEGG pathway; (b) orthologous groups between tea plant and other 11 representative plant species (e.g. TEA005863.1); (c) correlation analysis between gene expression and metabolite accumulation; (d) ORF finder tool; (e) polymorphic SSR discovery tool; (f) automatic primer designer; and (g) primer blaster (e.g. tea caffeine synthesis gene).

We built abundant of analytic tools for users to fully explore and/or analyse the rich omics‐data sets of tea plant in TPIA:

*Gene set functional enrichment*. An enrichment analysis tool was established to help users to fast and efficiently determine the functions of a given list of genes (Figure 6a). Users can perform gene functional enrichment analysis based on two function catalogs: GO term and KEGG pathway. The results returned the significantly enriched GO or KEGG functional categories that were further cross‐linked to the specific public databases.
*Orthologous groups*. An interface is designed for users to search orthologous groups between tea plant and other 11 representative plant species by using the gene identifiers (Figure 6b). The output displayed the details of orthologous groups and their phylogeny constructed using RAxML automatically.
*Correlation analysis*. This tool is designed to help users to investigate the correlations between expression levels of two given gene list (Gene2Gene) or correlation between the gene expression and accumulation of specific metabolites (Gene2Metabolite) (Figure 6c). Three methods that include ‘*pearson*’, ‘*kendall*’ and ‘*spearman*’ are adopted for correlation test. The user simply needs to input the gene list of interest and then select the corresponding calculation method to perform correlation analysis. The result will return their correlation coefficient and significance *P*‐value that meet the thresholds, which was further visualized using heatmap.
*Open reading frame finder*. This tool was designed to search open reading frames (ORFs) in the DNA/transcript sequence of interest. The results returned the range of each ORF, along with its protein translation. They can be further linked to blast for searching homologs in TPIA.
*Batch retrieve data*. This data‐mining tool is designed helping users to export custom data sets from TPIA. A web form allows users to input a list of gene identifiers to batch retrieve multiple types of sequences (cds, transcripts, exon, upstream and downstream sequence) and diverse expression data sets (eight tissues, biotic and abiotic stresses, leaves of close relatives) from TPIA.
*Automatic primer designer*. Primer designer was designed to help users to build primers that are specific to intended polymerase chain reaction experiments. Primer3 is used to generate the candidate primer pairs for a given template sequence (Untergasser *et al*., 2012). Another specific tool, Primer Blaster, was designed to test the specificity of any primer pair on the tea plant genome by using BLAST. The results returned a table that displays all tested primers with primer name and positions, location on scaffold, product size and number of hits on the tea plant genome.
*Polymorphic SSR discovery*. This tool was built to help users to massively detect polymorphic SSRs (PolySSR) in tea plant, an import and widely used genetic markers for tea plant population genetic study and molecular breeding. Our previously developed CandiSSR pipeline was deployed online to achieve this goal (Xia *et al*., 2016). Users can upload the assembled transcript sequences, or genomic sequences, or even organelle sequences to search polySSRs. The output was a table displaying the details of identified polySSR, including the SSR type, number of repeats, scaffold location, dispersion degree, missing rate, corresponding primer pairs and their transferability.

### A case application of TPIA provides new insights into phytochemical content variation of section *Thea* under a well‐resolved phylogenetic framework

**Figure 7:**
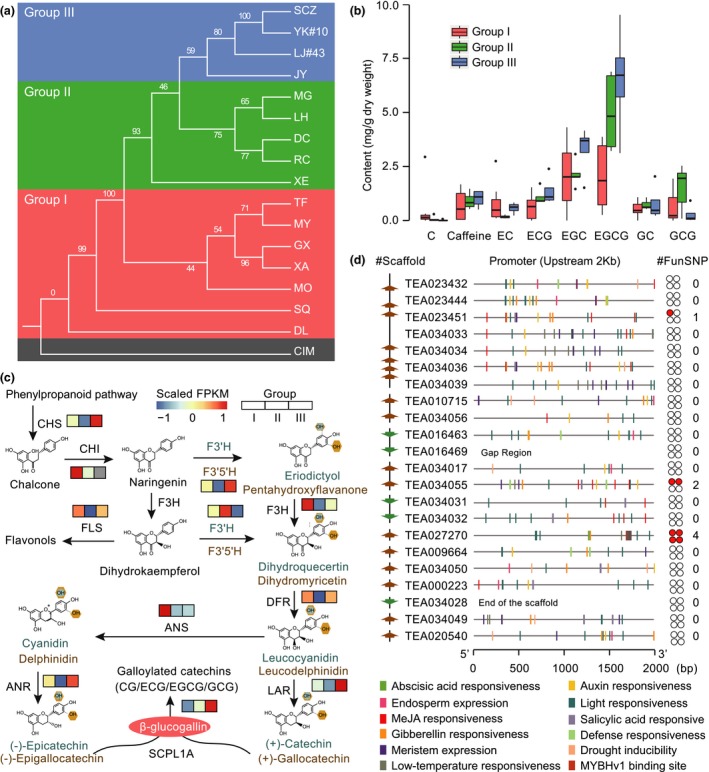
A case application of TPIA. (a) Phylogenetic tree of section *Thea* of genus *Camellia* constructed using 313 high‐quality 1:1 single‐copy orthologous genes from transcriptome data of TPIA; (b) accumulation dynamics of tea quality associated characteristic metabolites under a well‐resolved phylogenetic framework of section *Thea*; (c) expression pattern of key genes involved in catechins biosynthesis across different *Thea* groups. The FPKM values of expression levels are centred and scaled using ‘*pheatmap*’ package; (d) variation of 22 SCPL1A genes across different *Thea* species. The left panel indicates the scaffolds that hold the SCPL1A genes (brown: reverse strand; green: forward strand). The middle panel shows the *cis*‐regulatory elements identified in the putative promoter region (upstream 2 kb) of each SCPL1A gene. The right panel represents the total number of functional (nonsynonymous) SNPs detected in the coding sequences of each SCPL1A gene, which is further visualized by red solid circles.

Tea plant is a species from section *Thea* of genus *Camellia*. This section hosts several representative species that exhibit great economic importance in the production of global tea beverage (Ming and Bartholomew, 2007). However, due to the frequent interspecific hybridization and lacking of suitable nuclear genes for evolutionary analyses, the clear phylogenetic relationship of this section remains poorly understood, which hampers their efficient development and utilization towards tea plant modern breeding. To address this, we retrieve the transcriptome data of *Thea* species from TPIA and cluster them into 90 912 orthologous groups using OrthoMCL (Li *et al*., 2003). This is further analysed to generate a total of 313 high‐quality 1:1 single‐copy orthologous genes. The protein sequences of these single‐copy genes are individually aligned and concatenated to a super‐sequence to construct the phylogeny using RaxML package with *C. impressinervis* (CIM) selected as outgroup. Results show that the species from section *Thea* can be apparently divided into three groups (Figure 7a). As expected, the cultivated tea plants are clustered together (Group III) and sister to the group (Group II) that consists of *C. makuanica* (MG), *C. atrothea* (LH), *C. tachangensis* (DC), *C. pubescens* (RC) and *C. parvisepala* (XE). The *C. gymnogyna* (TF), *C. ptilophylla* (MY), *C. kwangsiensis* (GX), *C. angustifolia* (XA), *C. leptophylla* (MO), *C. tetracacca* (SQ) and *C. taliensis* (DL) are grouped into a single clade (Group I) with the DL is demonstrated to be the basal lineage of the section *Thea*. Most of the branches of the constructed phylogenetic tree are supported by ≥75% bootstrap values, indicating that the sufficient amount of low‐copy nuclear genes have natural advantages in solving the plant phylogeny (Zeng *et al*., 2014).

The well‐resolved phylogeny of section *Thea* allows us to further globally investigate the content variations of major characteristic metabolites that determine tea quality under an evolutionary framework. To archive this goal, we retrieve the accumulation data of catechins and caffeine in the leaves of plant species from section *Thea* and then mapped them onto the constructed phylogeny according to the species classification. The results show that the contents of tea quality associated metabolites (e.g. catechins) increase along with the species evolutionary trajectory, with the recent diverged tea plants accumulate more catechins and caffeine than species from the ancient clade (Figure 7b). The galloylated catechins, particularly ECG and EGCG, show the largest content differences. To the best of our knowledge, this is the first investigation on the evolutionary landscape of metabolites associated with tea quality in section *Thea*.

We also download the expression and variation data from TPIA to further investigate the genetic basis underlying evolution dynamics of metabolites in section *Thea*. We show that most of the key genes encoding enzymes involving in catechins biosynthesis, such as CHS, F3′5′H, ANR, LAR and SCPL1A, are highly expressed in the leaves of species from recent diverged clade (Group III) compared to other two groups (Figure 7c). Of them, the global expression patterns of *SCPL1A* genes are highly correlated with the catechins accumulations among the three groups. *SCPL1A* gene is previously evidenced to be involved in galloylation of flavan‐3‐ols (Wei *et al*., 2018); thus, the expression characteristics of *SCPL1A* genes would potentially facilitate the elucidation of the dynamic accumulation of catechin, particularly galloylated catechins, in leaves of different tea plant species. As totally 22 *SCPL1A* genes located in tea plant genome (Wei *et al*., 2018), we further examine their single nucleotide polymorphisms (SNP) across *Thea* species using the variation data from TPIA. Results show that only three copies (TEA023451, TEA034055 and TEA027270) possess few nonsynonymous mutations, indicating the conservation of SCPL genes in *Thea* species (Figure 7d). Nevertheless, we find that the putative promoter regions (upstream 2 kb) of the 22 *SCPL1A* genes are quite complex, which harbour several development and stress‐related regulatory elements, including abscisic acid (ABA) responsive element, auxin responsive element, defence responsiveness, drought inducibility, low‐temperature responsive element and MYB binding site (Figure 7d). Taken together, we apply TPIA by integrating transcriptomic, phylogenetic, metabolic and genomic data to globally uncover the dynamic evolutions of tea quality‐related characteristic metabolites in section *Thea*, providing new insights and essential clues for future tea plant functional genomics studies and breeding.

## Conclusions

In summary, we have built a comprehensive knowledge database for tea plant. It contains abundant genomic, transcriptomic, metabolic and epigenetic data as well as extensive germplasm resources. A large number of versatile analytic tools (e.g. JBrowse, blast, correlation analysis, metabolic pathway, GO/KEGG enrichment and PolySSR) were launched to establish their inner network to help users performing comparative, evolutionary and functional analysis. In the seeing further, we will further closely collaborate with worldwide research groups and incorporate more flexible tools and novel publicly available resources, such as genomic data from close species, genetic variation and morphological data from populations, into TPIA as immediately as they are available. Through timely and long‐term maintenance, we hope TPIA can be a central gateway for tea community to better understand the biology of tea plant that benefits the whole tea industry.

## Funding information

This work was supported by the National Key Research and Development Program of China (2018YFD1000601), the National Natural Science Foundation of China (31800180), the Science and Technology Project of Anhui Province (13Z03012), the Special Innovative Province Construction in Anhui Province (15czs08032), the Special Project for Central Guiding Science and Technology Innovation of Region in Anhui Province (2016080503B024), the China Postdoctoral Science Foundation (No. 2017M621992) and the Postdoctoral Science Foundation of Anhui Province, China (No. 2017B189).

## Author contributions

E.‐H.X., Z.‐Z.Z, C.‐L.W. and X.‐C.W. designed the project; E.‐H.X. constructed the website; E.‐H.X., F.‐D.L., W.T., Q.W., H.‐J.Z., G.‐R.H. and P.‐H.L. collected the data; E.‐H.X. wrote the paper; E.‐H.X. and W.T. revised the paper.

## Conflict of interest

The authors declare that they have no competing interests.

## Supporting information


**Figure S1** Analysis of SSRs in tea plant genome.
**Figure S2** Analysis of repeat sequences in tea plant genome.
**Figure S3** Abundant and well organized functionally characterized genes of tea plant.
**Figure S4** Collection and utilization of tea plant transcriptomes.
**Figure S5** Rapid retrieval of germplasm information worldwide.
