# Supplementary material for: Tea Plant Information Archive: a comprehensive genomics and bioinformatics platform for tea plant
Source: Plant Biotechnol J. 2019 Apr 11;17(10):1938–53. doi: 10.1111/pbi.13111 (PMC6737018; doi:10.1111/pbi.13111)
Supplement: Supplementary file 1 — Figure S1 Analysis of SSRs in tea plant genome. Figure S2 Analysis of repeat sequences in tea plant genome. Figure S3 Abundant and well organized functionally characterized genes of tea plant. Figure S4 Collection and utilization of tea plant transcriptomes. Figure S5 Rapid retrieval of germplasm information worldwide. [file PBI-17-1938-s001.pdf]

Additional Files

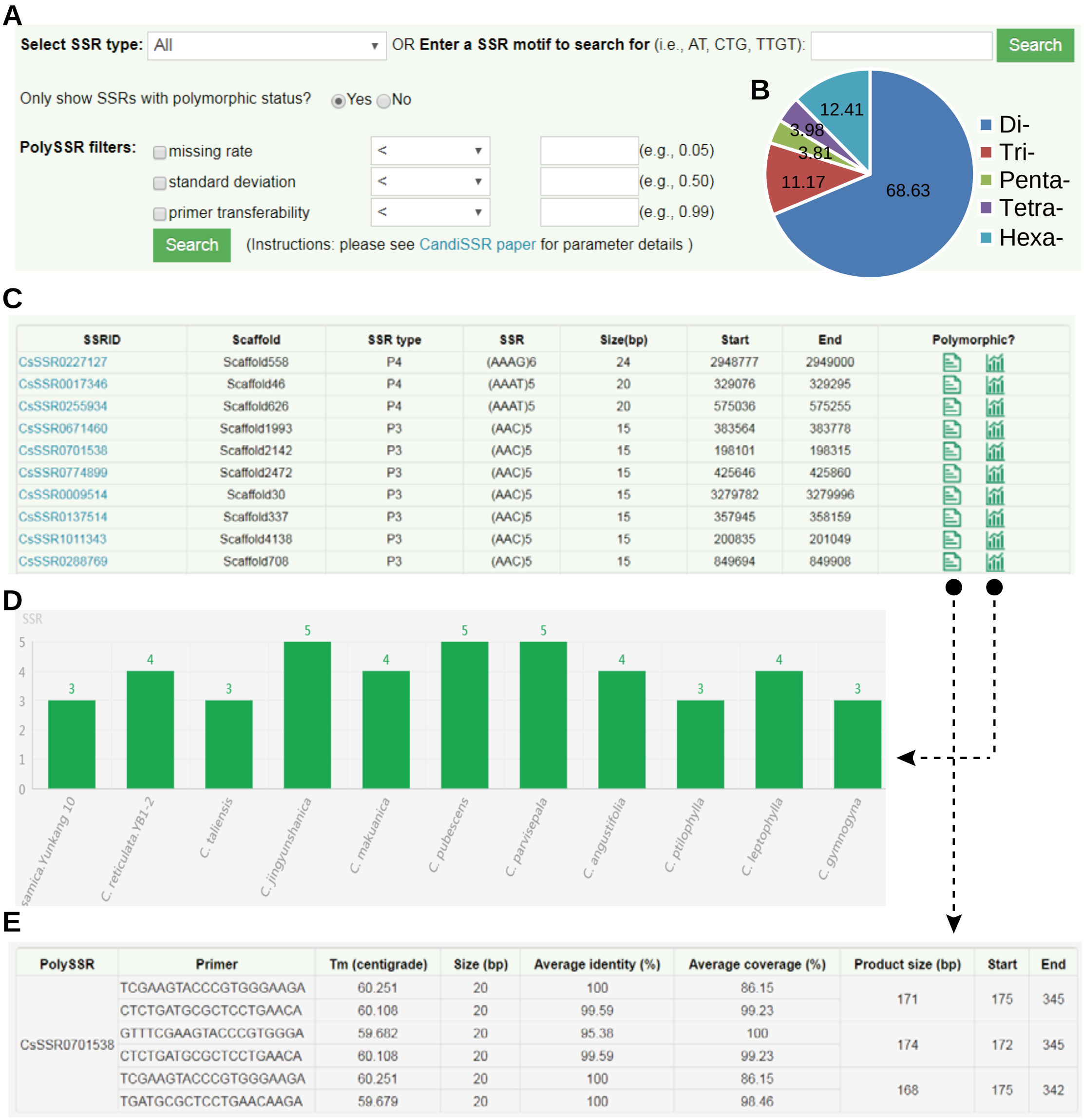

SSRID	Scaffold	SSR type	SSR	Size(bp)	Start	End	Polymorphic?
CsSSR0227127	Scaffold558	P4	(AAAG)6	24	2948777	2949000	
CsSSR0017346	Scaffold46	P4	(AAAT)5	20	329076	329295	
CsSSR0255934	Scaffold626	P4	(AAAT)5	20	575036	575255	
CsSSR0671460	Scaffold1993	P3	(AAC)5	15	383564	383778	
CsSSR0701538	Scaffold2142	P3	(AAC)5	15	198101	198315	
CsSSR0774899	Scaffold2472	P3	(AAC)5	15	425646	425860	
CsSSR0009514	Scaffold30	P3	(AAC)5	15	3279782	3279996	
CsSSR0137514	Scaffold337	P3	(AAC)5	15	357945	358159	
CsSSR1011343	Scaffold4138	P3	(AAC)5	15	200835	201049	
CsSSR0288769	Scaffold708	P3	(AAC)5	15	849694	849908	

PolySSR	Primer	Tm (centigrade)	Size (bp)	Average identity (%)	Average coverage (%)	Product size (bp)	Start	End
CsSSR0701538	TCGAAGTACCCGTGGGAAGA	60.251	20	100	86.15	171	175	345
	CTCTGATGCGCTCCTGAACA	60.108	20	99.59	99.23			
	GTTTCGAAGTACCCGTGGGA	59.682	20	95.38	100	174	172	345
	CTCTGATGCGCTCCTGAACA	60.108	20	99.59	99.23			
	TCGAAGTACCCGTGGGAAGA	60.251	20	100	86.15	168	175	342
	TGATGCGCTCCTGAACAAGA	59.679	20	100	98.46			

Figure S1 Analysis of SSRs in tea plant genome. (A) SSR selection; (B) SSR statistic; (C) Search result of specific SSRs; (D) SSR Polymorphism among 11 representative tea plant species; and (E) Three primer pairs designed for each polymorphic SSR.

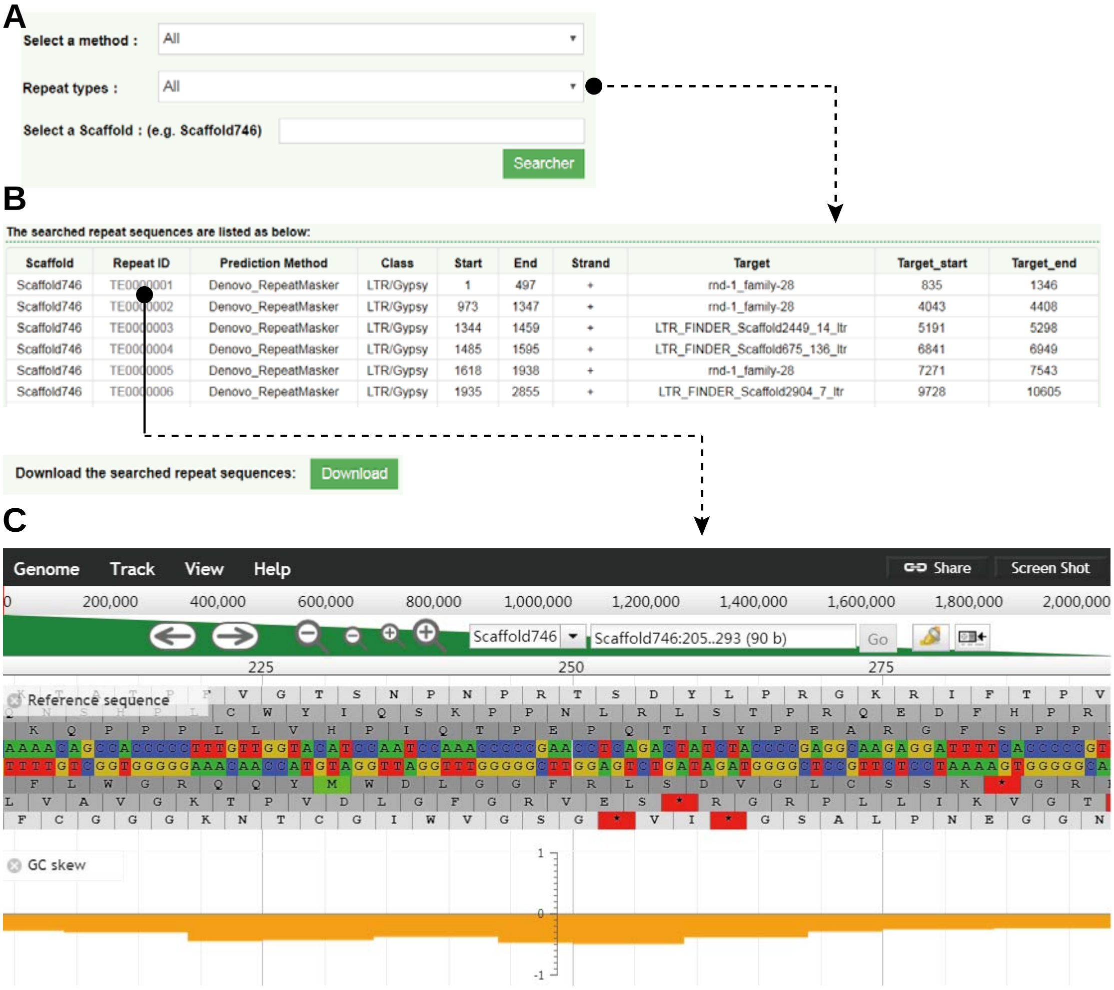

Figure S2 Analysis of repeat sequences in tea plant genome. (A) Annotation method, repeat type and scaffolds selection; (B) Search result of specific repeat sequences with download; and (C) Visualization of repeat sequences using JBrowser.

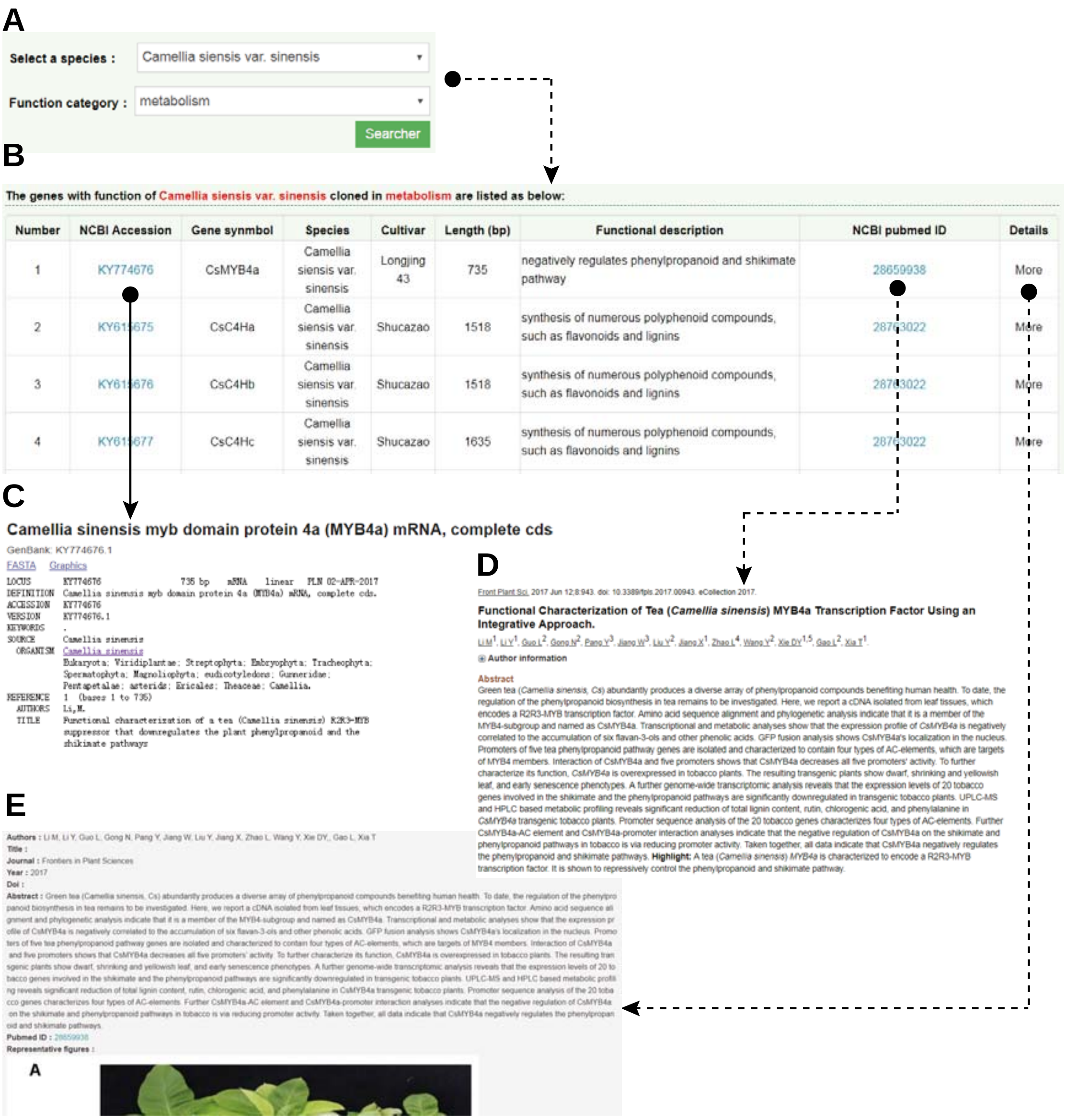

Figure S3 Abundant and well organized functionally characterized genes of tea plant. (A) Species and functional category selection; (B) Search result of metabolism related genes in *Camellia sinensis* var. *sinensis*; (C) Sequence records in NCBI nucleotide database; (D) Related reference in PubMed; and (E) Detail information of the collected genes, particularly the representative figures of functional experiments.

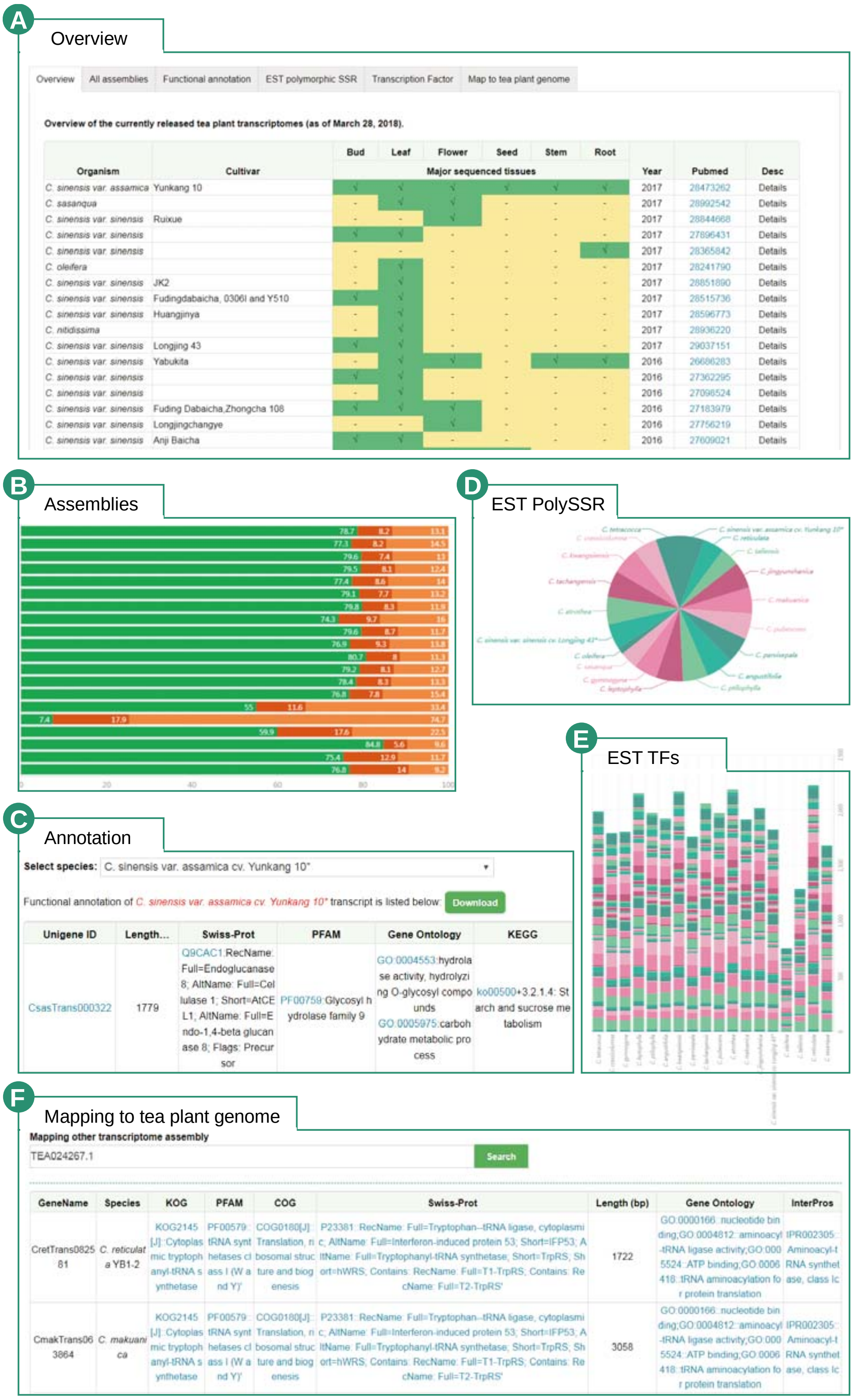

Figure S4 Collection and utilization of tea plant transcriptomes. (A) Overview of the transcriptome collection; (B) Assemblies; (C) Functional annotations, (D) EST polymorphic SSRs, (E) EST transcription factors, and (F) Mapping the transcripts to tea plant genome.

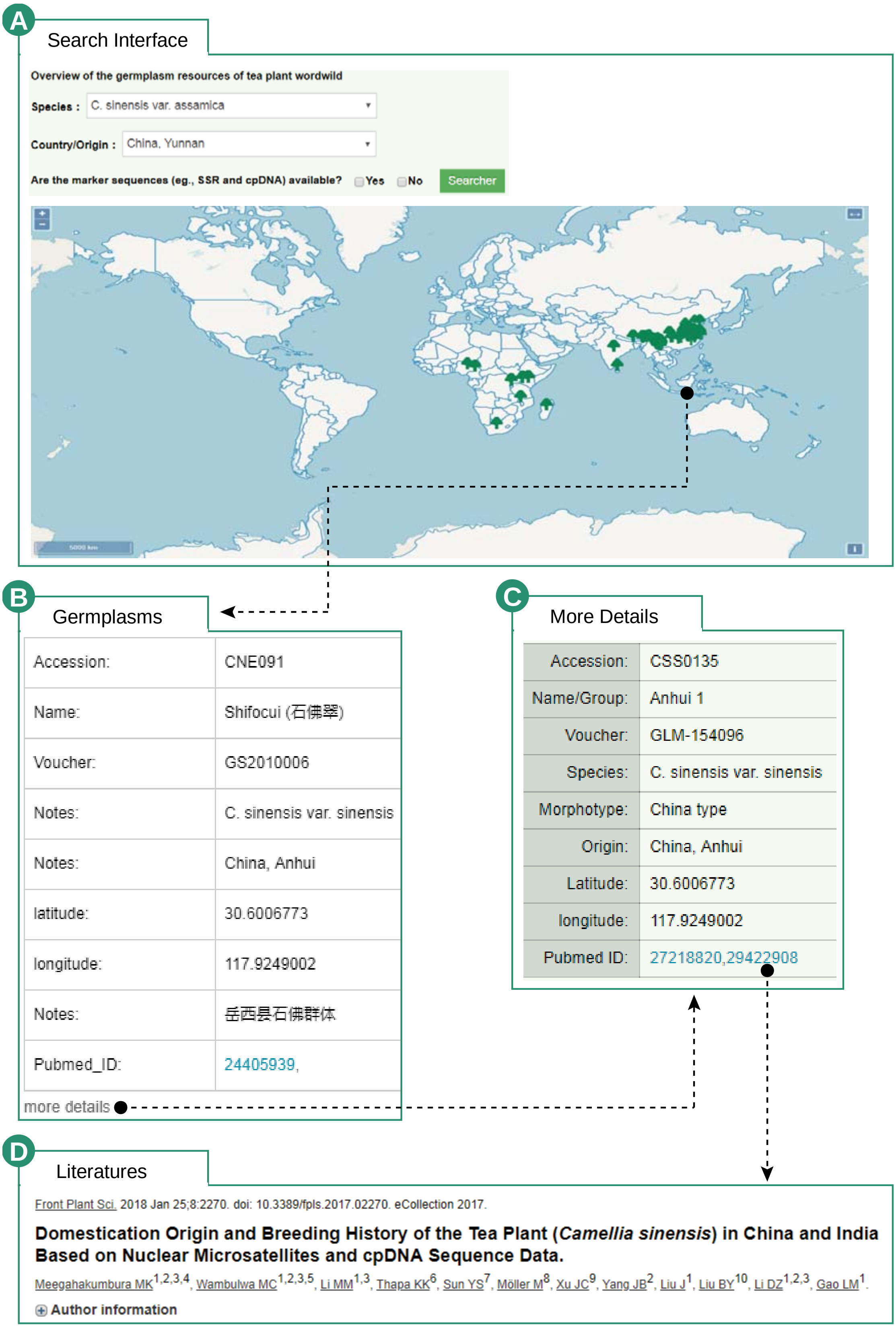

Figure S5 Rapid retrieval of tea plant germplasm information. (A) Search interface; (B) Searched germplasms; (C) Details of the search results; and (D) Related literatures.
